# Blind recognition of channel codes based on dual-branch feature fusion convolutional neural networks

**DOI:** 10.1038/s41598-026-35558-7

**Published:** 2026-01-13

**Authors:** Yuwei Ma, Yingke Lei, Changming Liu, Wei Wang, Fei Teng, Chuang Peng, Hu Jin, Hui Feng, Mengbo Zhang, Yu Pan

**Affiliations:** 1 College of Electronic Engineering, National University of Defense Technology, Hefei, 230037 China; 2 Tongfang Electronic Technology Co., Ltd, Jiujiang, 332000 China; 3 National Key Laboratory of Electromagnetic Space Security, Jiaxing, 314000 China

**Keywords:** Channel codes, Blind identification, Dilated convolution, Multidimensional features, Non-cooperative system, Engineering, Mathematics and computing

## Abstract

Facing heterogeneous signals increasing in dynamic spectrum, cognitive radio urgently needs blind channel coding identification. This technology addresses the core challenge of unknown coding schemes in non-cooperative communications. Existing methods are typically restricted to specific coding types and suffer from poor identification accuracy and robustness. To mitigate this constraint, we propose a Dual-Branch Feature Fusion Convolutional Neural Network (DBFCNN) framework for fine-grained identification among seven common channel-coding schemes. The network adopts a two-branch architecture. One branch employs multi-scale dilated convolutions to extract long-range dependencies in the received bit sequence, the other is a statistical branch that extract descriptors such as run length, entropy values, coding depth and so on to expose code-specific algebraic characteristics. The fused representation is fed to a fully connected classifier to jointly identify the seven code types. Extensive simulations demonstrate that DBFCNN improves identification accuracy by about 5% (absolute) over a strong prior baseline under comparable settings, proving the feasibility and effectiveness of the method.

## Introduction

In cognitive-radio and dynamic-spectrum-access systems, secondary users share licensed bands opportunistically to boost spectral efficiency. This sharing is only possible if the radios can autonomously sense and adapt to an unknown wireless environment. In practice, primary users select different channel codes according to varying channel states and services. Consequently, cognitive receivers have no prior knowledge of the coding parameters, and no control channel is available to supply them. In this context, blind channel-coding recognition strengthens the cognitive cycle by letting the secondary user extract the code types and parameters of an unknown link. Reliable decoding can then proceed, enabling robust connectivity. Therefore, a low-complexity, resilient coding-type identifier is a prerequisite for pushing cognitive-radio performance beyond today’s limits and for raising spectrum utilization and link reliability in heterogeneous, non-cooperative networks^1^.

Channel codes can be broadly classified into two main types: linear block codes and convolutional codes. Linear block codes are further subdivided to include Hamming, Bose–Chaudhuri–Hocquenghem (BCH), Low-Density Parity-Check (LDPC), Reed-Solomon (RS), and Polar codes, while convolutional codes include both traditional convolutional codes and Turbo codes. Although parameter-estimation techniques for specific codes such as linear block codes^2–4^, BCH codes^5–7^, RS codes^8–10^, LDPC codes^11–13^, Turbo codes^14,15^, and convolutional codes^16–18^ have attained considerable maturity, investigations into the blind identification of coding type remain comparatively scarce.These methods, however, assume that the code class is already known, and they focus on estimating length, rate or generator/parity matrices. Surveys by LIU[24] reveal that joint, fine-grained identification of multiple code classes–without any prior–has received comparatively little attention: only a handful of studies target more than three codes simultaneously, and their average accuracy remains below 80% at $BER=10^{-2}$.

In recent years, deep-learning-based approaches have yielded significant advances in both code-type and parameter identification^19–22^. Owing to their powerful nonlinear feature-extraction capabilities, these methods can effectively handle the complex real-world communication data^23^. Nevertheless, existing studies predominantly rely on either hand-crafted features (such as entropy, run-length, code-weight and so on) or global codeword representations, thereby enabling only coarse-grained discrimination among two or three specific code types. A fine-grained classifier able to distinguish seven commonly used codes (Hamming, BCH, LDPC, RS, Polar, convolutional, Turbo) in one shot is still lacking. The few existing algorithms that do target multi-type identification exhibit recognition accuracy that require further improvement.

To bridge this gap, we propose the Dual-Branch Feature-Fusion Convolutional Neural Network (DBFCNN). In Branch 1, the raw bit stream is segmented into 8-bit words and processed by multi-scale, multi-dilation convolutions to capture structural attributes such as constraint length and parity density. Branch 2 computes hand-crafted descriptors–namely, normalized entropy, run-length vector, and code-weight spectrum–and feeds them through a lightweight fully-connected sub-network to obtain global codeword representations. A late-fusion layer concatenates both feature vectors, learns fine-grained inter-codeword dependencies, and performs the final classification. Simulation results demonstrate that this approach significantly improves the identification accuracy of mixed channel-coding types.

The main contributions of this paper are summarized as follows:

1. Building upon embedding segmentation mechanism–i.e., splitting the raw bit stream into fixed-length words that are subsequently mapped to dense vectors–we integrate multi-scale convolutional kernels ($k=3,5,7$) with varying dilation rates ($d=1,2,5$). This design significantly expands the receptive field while maintaining parameter efficiency. Multi-scale convolution extends receptive coverage through kernel diversity without increasing parameter count, while dilated convolution captures both dense local features and sparse global correlations by modulating sampling granularity rather than simply enlarging the window size.
2. We propose a unique dual-branch architecture combining data-driven and handcrafted feature extraction. This proposed approach provides complementary information for channel coding identification, simultaneously overcoming two limitations: the poor bit-error-rate (BER) robustness of purely data-driven networks and the low classification accuracy of manual feature extraction.
3. The DBFCNN method is compared with previous studies. Extensive experiments demonstrate that, at a BER below $10^{-3}$, the DBFCNN achieves an average recognition accuracy of 98% across seven coding types, surpassing the best-performing Multi-Scale Dilated Convolutional Neural Network (MSDCNN)^24^ by 10.87%. Moreover, the overall recognition accuracy rate for each type of channel coding reaches 95%, providing a solution for blind recognition in complex channel environments.

Subsequent sections present the paper’s organization: Section “Related work” surveys foundational research; Section “Problem formulation” establishes a mathematical framework for blind channel-coding identification; Section "The proposed approach" details the proposed DBFCNN framework, which adopts a dual-branch parallel structure to separately process the original bit sequence and the global statistical features extracted from hand-crafted descriptors, respectively, and achieves type identification after branch fusion; Section “Experimental results” conducts comprehensive simulations and performance comparisons; Section “Conclusion” concludes the paper and outlines directions for future research.

## Related work

Existing approaches to channel-coding type identification can be broadly classified into conventional methods and deep learning methods. Conventional methods are generally hampered by excessive computational complexity, limited adaptability to diverse coding schemes, and an inability to fully exploit large-scale datasets.Swaminathan et al.^25^ distinguish linear block codes from convolutional codes by computing the rank ratio of the received signal matrix, but their method cannot further discriminate among codes that belong to the same family.

Deep learning, by contrast, provides powerful non-linear feature extraction and opens a new route toward joint, fine-grained recognition of multiple code classes. Within the deep-learning branch, some studies exploit the similarity between coded bit streams and natural-language sentences. Qin et al.^26^ mapped encoding sequences to word vectors and employed TextCNN to perform type recognition. Building on this, Li et al.^27^ employed a TextCNN embedding segmentation mechanism to transform the recognition problem into a natural language processing (NLP) task, thereby achieving classification of linear block codes, convolutional codes, and Turbo codes. However, due to limitations in model capacity, TextCNN encounters challenges in scaling to scenarios involving the joint recognition of multiple code types.

Beyond pure TextCNN schemes, richer network architectures have been successively investigated. Yang et al.^28^ introduce a recurrent CNN (RCNN) with feedback loops to capture long-range dependencies within convolutional codewords, achieving 1.8% higher accuracy than a vanilla CNN at BER=$10^{-3}$; however, only a single code type is recognised. Huang et al.^29^ combine bidirectional long short-term memory (BiLSTM) with CNN and effectively identify three different channel codes. To improve fault tolerance, MEIF et al.^30^ modify the traditional recurrent neural network (RNN); the enhanced net successfully recognises four distinct code types.

Meanwhile, hybrid methods based on multi-dimensional feature extraction are also increasingly being applied in the identification of code type. Peng et al.^31^ proposed a unified framework that fuses multi-dimensional features with an efficient channel attention module (ECA-Net), enabling the joint estimation of code type, length, and rate via randomness analysis and deep convolutional networks. Pengcheng Laboratory proposed a generalized finite-field Fourier transform (GFFT) feature extraction method, combining artificial features such as code weight, entropy value, and run length to construct an Euclidean distance classifier. Despite their ability to capture intrinsic code characteristics, these multi-dimensional approaches suffer from issues such as exponential growth in feature dimensionality and degraded generalization under high BER conditions.

Additionally, to meet the demand for multi-scale perception, LIU et al.^24^ designed the MSDCNN, which embeds parallel dilated kernels to enlarge the receptive field without expanding the parameter count, thereby improving recognition accuracy across seven prevalent coding schemes. Nevertheless, the kernel design overlooks the algebraic periodicity inherent in linear block codes, resulting in room for improvement in recognition accuracy and discrimination for LDPC codes, Polar codes, and RS codes.

In summary, channel-coding-type recognition based on deep learning has attracted considerable attention as a promising paradigm; however, existing approaches have yet to achieve a balance between model generalization, feature discriminability and computational efficiency. Consequently, an innovative architecture that tightly integrates algebraic structures with deep learning is imperative. To this end, the DBFCNN proposed in this paper unifies encoding-sequence vectorized mapping, low-complexity algebraic features, multi-scale dilated sensing, and cross-branch feature aggregation. Detailed design principles and implementation are presented in Section "The proposed approach".

## Problem formulation

**Fig. 1:**
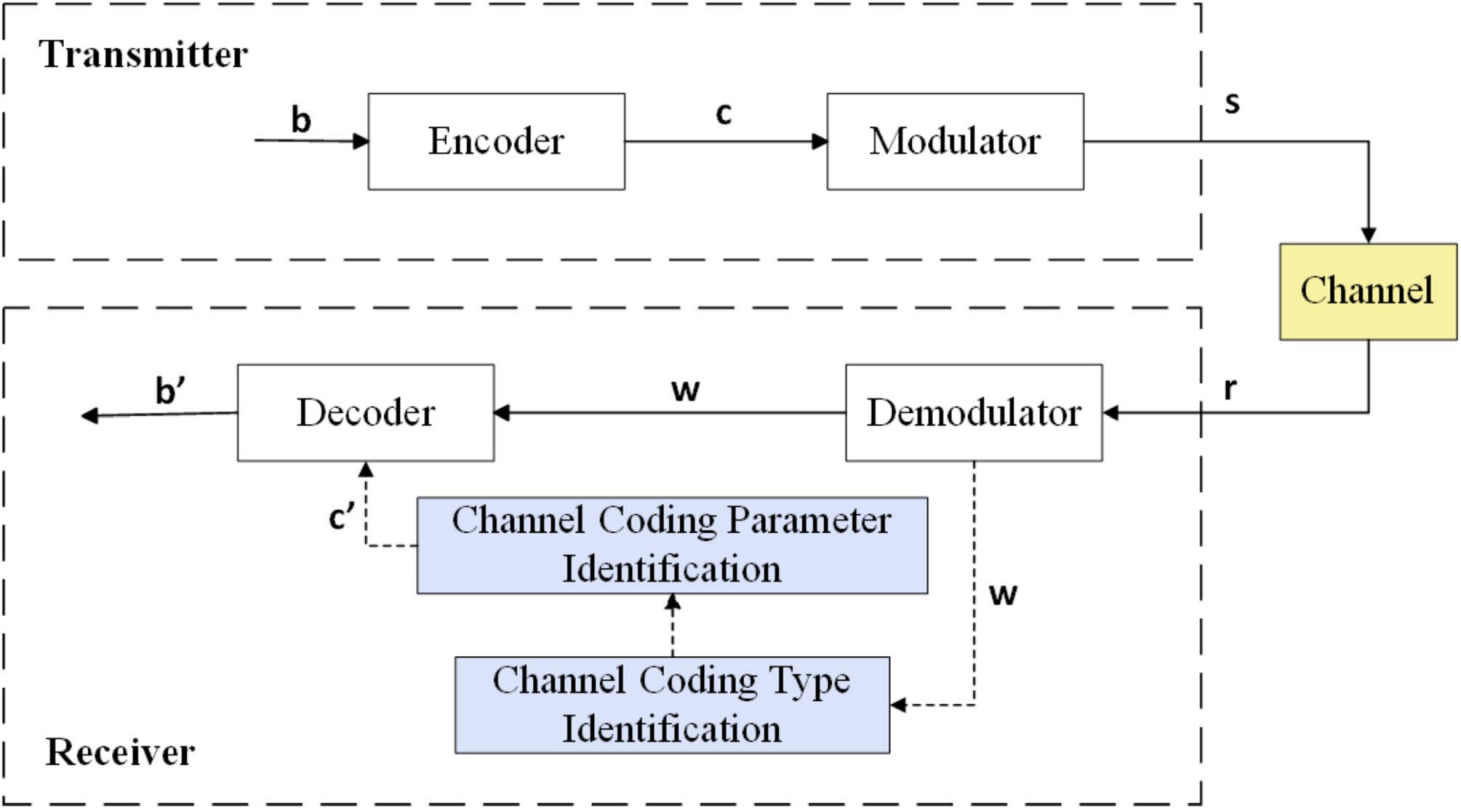
Channel code type recognition process.

In non-cooperative communications, blind identification of channel codes is the process of inferring the transmitter’s encoding scheme from intercepted symbols without prior knowledge. A canonical communication system model is illustrated in Fig. 1. Within this model, the blind identification task considered herein is situated at the receiver, immediately after demodulation and prior to decoding^32^.

Owing to noise, fading, loss, etc. introduced by the channel, the binary sequence *w* obtained after preprocessing steps such as demodulation of the intercepted signal *r* can be regarded as an erroneous realization of the codeword *c* generated by the transmitter’s channel encoder, i.e.,

1 $$\begin{aligned} {\textbf {w}} = {\textbf {c}} \oplus {\textbf {e}} \end{aligned}$$

where *e* denotes the additive error vector, and $\oplus$ the bit-wise XOR operation. The channel code employed at the transmitter is unknown and is assumed to belong to a finite candidate set$\Theta =\bigl \{\theta _{\text {Ham}}, \theta _{\text {Conv}}, \theta _{\text {Turbo}}, \theta _{\text {BCH}}, \theta _{\text {LDPC}}, \theta _{\text {RS}}, \theta _{\text {Polar}}\bigr \}$. Each code type imposes a specific algebraic structure on *w*. Blind identification therefore reduces to inferring the mapping $\mathscr {F}: \{0,1\}^{L} \rightarrow \Theta$ that recovers the underlying code type by exploiting the intrinsic structural disparities among codeword spaces in the presence of channel errors, which is

2 $$\begin{aligned} \mathscr {F}(\textbf{w}) = \arg \max _{\theta \in \Theta } P (\textbf{w} \in \mathscr {V}_{\theta }) \end{aligned}$$

where *L* is the length of the sequence, $\mathscr {V}_{\theta }$ denotes the typical codeword space associated with the $\theta$-type code (Table 1). Accordingly, the identification task is formulated as a composite hypothesis-testing problem.

**Table 1:** Typical codeword space for channel coding.

Code type	Codeword space	Algebraic essence
Linear-Block-Codes	$\langle G\rangle \subset \mathbb {F}_{2}^{n}$	*k*-dim vector subspace of $\mathbb {F}_{2}^{n}$
BCH-Codes	$\mathscr {J}(g(x)) \cap \mathbb {F}_{2}^{n}$	Ideal in GF $\mathbb {F}_{2^{m}}[x]/(x^{n}-1)$
RS-Codes	$\textrm{ev}_{\alpha }(\mathscr {P}_{d}) \subset \mathbb {F}_{q}^{n}$	Reed-Solomon evaluation mapping
LDPC-Codes	$\ker (H)$	Null space of sparse *H* matrix
Convolutional-Codes	$\bigcup _{t}\phi (s_{t})$	Trellis paths $\phi :\mathscr {S} \rightarrow \mathbb {F}_{2}$
Turbo-Codes	$C_{1} \bowtie _{\pi } C_{1}$	Concatenated subcodes via interleaver
Polar-Codes	$\textrm{span}\{g_{i}:i\in \mathscr {A}\} \subset \mathbb {F}_{2}^{n}$	Channel polarization: $\textbf{G}_{N} = \textbf{B}_{N} \cdot \textbf{G}_{2}^{\otimes n}$

Let the BER of the sequence *w* over sequence *c* denoted by *p*. The blind identification problem is then cast as a composite hypothesis-testing problem in which is treated as an unknown nuisance parameter:

3 $$\begin{aligned} \mathscr {H}_{k}: \theta = \theta _{k},\ p \in \left( 10^{-5}, 10^{-1}\right) \end{aligned}$$

where $$k=\{Ham,Conv,\cdots ,Polar\}$$.

A generalized likelihood ratio is used for decision making:

4 $$\begin{aligned} \hat{\theta } = \arg \max _{\theta _{k} \in \Theta } \, \sup _{p \in (10^{-5}, 10^{-1})} \mathscr {L}(\theta _{k}, p; \textbf{w}) \end{aligned}$$

where $\mathscr {L}\left( \theta _{k}, p;\textbf{w}\right) = \mathbb {P}\left( \textbf{w}\mid \theta _{k}, p\right)$ is the likelihood function. Since direct evaluation of the likelihood function entails a high-dimensional summation and is contingent upon the unknown parameter *p*, a code-structure invariant $\mathscr {J}_{\theta _{k}}$ is introduced. This invariant maps the raw bitstream to low-dimensional feature vectors $\textbf{f} = \mathscr {T} \left( \textbf{w}, \mathscr {J}_{\theta _{k}} \right) \in \mathbb {R}^\textbf{d}$ that satisfy the *p*-invariance condition:

5 $$\begin{aligned} \frac{\partial }{\partial p} D_{KL} \left( \mathbb {P}(\textbf{f} \mid \theta _{i}) \parallel \mathbb {P}(\textbf{f} \mid \theta _{j}) \right) = 0,\, \forall \, i,j \end{aligned}$$

Consequently, the learned feature mapping $\mathscr {F}_{k}(\textbf{w})$ for each code type is inherently robust to the unknown bit-error rate *p*. By extracting this mapping from the underlying code structure via a deep-learning model, the classifier is able to discriminate among different coding types on the basis of the resulting invariant features.

## The proposed approach

In this section, a dual-branch deep neural network is devised for channel coding type identification. The architecture employs two parallel pathways: Branch 1 processes raw integer sequences through embedding and convolutional layers to extract local spatial correlations in bitstreams. Branch 2 incorporates handcrafted feature vectors, providing statistical properties with explicit physical interpretations. This dual-path design enables independent optimization of spatial distribution features (Branch 1) and algebraic attributes (Branch 2) prior to fusion, enhancing feature discriminability.

Critically, the branches exhibit complementary strengths. Branch 1 adaptively learns complex patterns but shows limited robustness at high BER, while Branch 2 maintains strong noise immunity yet has lower classification accuracy. The dual-branch architecture combines these advantages, preserving interpretability and noise resistance while boosting recognition accuracy through learned representations.

The detailed architecture follows.

### The structure of DBFCNN

The proposed DBFCNN adopts a heterogeneous feature-fusion architecture that comprises two parallel processing branches.

Branch 1, termed the *sequence-semantic branch*, accepts a one-dimensional integer sequence of length *L* obtained by *g*-bit grouping of the raw bit stream. An embedding layer first projects each integer into a 128-dimensional semantic space. Subsequently, features are extracted through multi-scale convolutions combined with multi-dilatation rate convolutional layers. The resulting feature maps are aggregated into a fixed 128-dimensional representation via global max-pooling, yielding a compact yet expressive descriptor that captures local spatial dependencies inherent to diverse coding structures.

Branch 2, termed the *algebraic-feature branch*, ingests a multidimensional handcrafted feature vector comprising run-length statistics, entropy, autocorrelation, coding depth, block features, linear features and spectral features. These descriptors are first transformed via a fully-connected layer with nonlinear activation, followed by batch normalization and dropout to mitigate overfitting and enhance generalization performance.

**Fig. 2:**
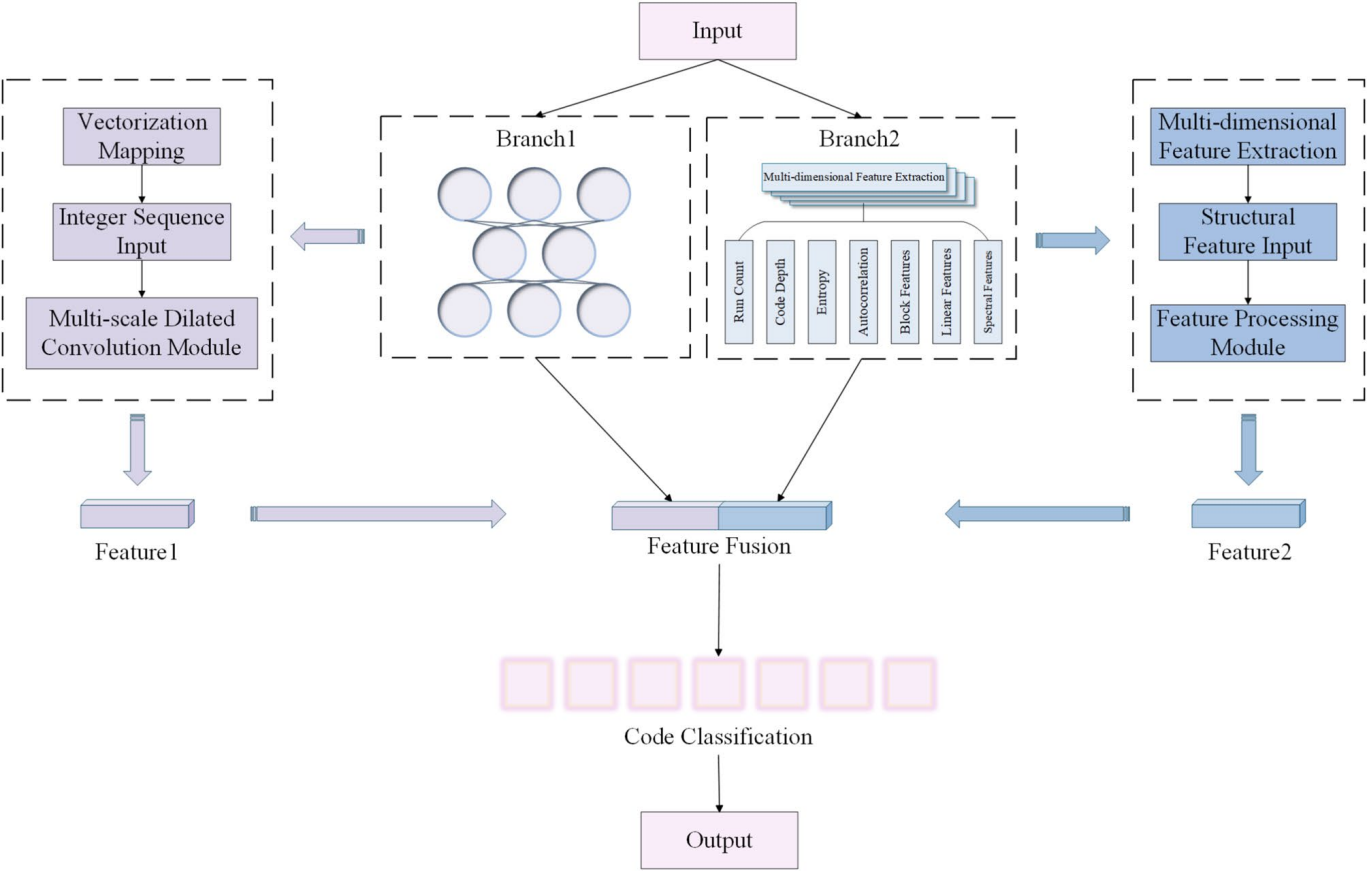
The structure of DBFCNN.

The outputs of these two branches are concatenated and forwarded to a fully-connected layer; batch normalization and dropout are subsequently applied to suppress overfitting. Finally, a softmax layer produces the posterior probability distribution over the seven candidate coding schemes. The complete architecture is depicted in Fig. 2.

### The coding sequence-semantic branch

**Fig. 3:**
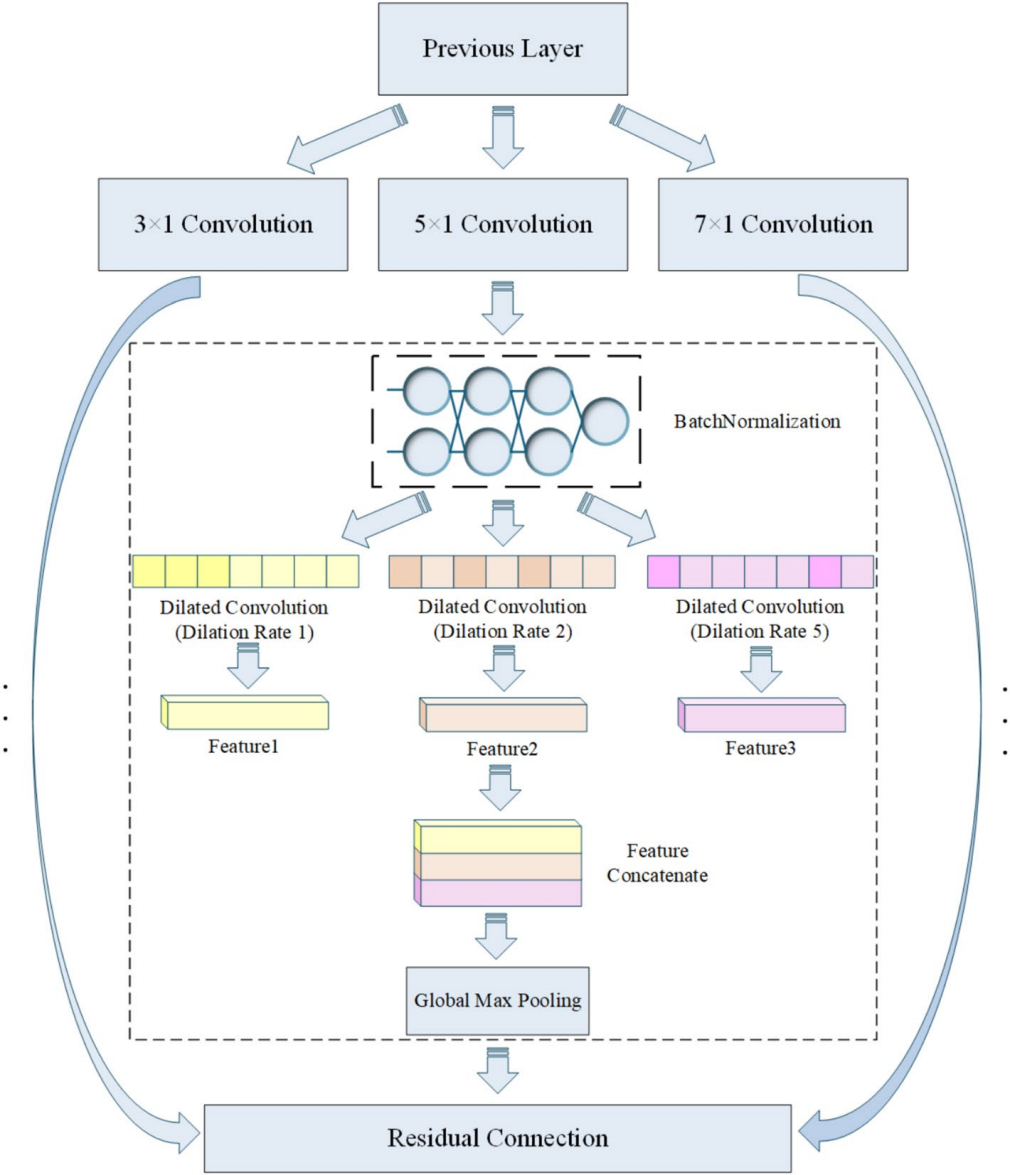
The structure of sequence-semantic branch.

To capture the long-range structural properties inherent to channel codes, this paper employs a multi-scale feature extraction strategy to achieve deep representation learning of encoded sequences; the corresponding architecture is illustrated in Fig. 3. The input layer receives the intercepted bit stream ${\textbf {w}} \in \{0,1\}^{L}$, which is first partitioned into *g*-bit groups^33^ to yield an integer sequence of length *L*. The selection of grouping bit count *g* balances computational efficiency and feature representation. Our architecture enables compatibility with varying *g* values. Section "The impact of semantic segmentation length on performance" details optimization strategies and experimental validation. Consequently, an (*L*/*g*)-dimensional discrete vector $\textbf{x}_{\textrm{int}} \in \mathbb {Z}^{L / g}$ is produced with a value domain of $\left[ 0, 2^{g} - 1\right]$.

6 $$\begin{aligned} \textbf{x}_{\textrm{int}}[i] = \sum _{k=0}^{g-1} w_{gi + k} \cdot 2^{k}, \quad 0< i < L/g \end{aligned}$$

A trainable embedding matrix $\textbf{E} \in \mathbb {R}^{128 \times 128}$ projects each integer token to a dense vector $h_{\text {embed}}[i] = \textbf{E}\left[ \textbf{x}_{\text {int}}[i]\right] \in \mathbb {R}^{128}$, where the embedding dimension $dim = 128$ is selected via grid-search optimization. The resulting tensor $\textbf{H}_{\text {embed}} \in \mathbb {R}^{(L / g) \times 128}$ thereby preserves the temporal ordering inherent in the input sequence.

This is followed by a multi-scale convolutional layer comprising three parallel branches with kernel sizes of 3, 5, and 7, respectively, while keeping all other hyperparameters consistent. The empirical validation demonstrates that this kernel combination provides robust performance across diverse coding structures and varying BER conditions. Ablation studies (Section "Experimental validation of multi-scale feature extraction effectiveness" details experimental validation.) confirm that removing any single branch results in significant performance degradation, underscoring the essential complementarity of these multi-scale features. Each branch is subsequently connected to a set of convolutional layers with multiple dilation rates to further expand the receptive fields while maintaining computational efficiency.

The multi-dilation convolutional layer employs a parallel three-branch structure to extract encoding-specific features at varying scales^34^. Each branch targets distinct structural characteristics. Dilation rate $m=1$ branch captures short-range constraints (suitable for the compact structure of linear block codes). $m=2$ branch extracts medium-range state transitions (suitable for convolutional codes). $m=5$ branch models long-range correlations (suitable for Turbo and LDPC codes). This combination of dilation rates captures both dense local and sparse global features, spanning short- to long-range interactions. It thereby enables simultaneous analysis of heterogeneous structures across diverse encoding types.

Each branch follows Equation 7 implementation with shared convolution kernels but distinct dilation rates. Left-padding maintains temporal invariance. The GELU (Gaussian Error Linear Unit) activation function provides smooth nonlinearity. The outputs of three branches are concatenated along the channel dimension to yield a 384-dimensional semantic feature vector $\textbf{v}_{\text {seq}} = \operatorname {Concat}\left( \textbf{v}^{(1)}, \textbf{v}^{(2)}, \textbf{v}^{(5)}\right) \in \mathbb {R}^{384}$. Each subspace corresponds to distinct structural scales. Global max-pooling then extracts peak activation features per channel.

7 $$\begin{aligned} \textbf{H}_{m} = \operatorname {GELU}\left( \operatorname {Conv1D}_{k=3,5,7}^{d=m}\left( \textbf{H}_{\text {embed}}\right) \right) \in \mathbb {R}^{(L/g) \times 128} \end{aligned}$$

Finally, the output features from the three multi-scale branches are concatenated with the residual path to form the final output of the semantic stream for the encoded sequence.

The specific parameter configuration of the coding sequence-semantic branch is shown in Table 2. Multi-scale 1D convolutional layers and dilated convolutional layers with varying dilation rates are employed to systematically extract heterogeneous structural features from sequences. The dilated convolutional branches share convolutional kernel parameters and maintain identical structures, differing only in dilation rates. This design ensures model simplicity while achieving multi-scale receptive field coverage, enhances the model’s ability to represent diverse coding structures, and provides robust feature support for classification, thereby effectively improving the accuracy of code type recognition.

**Table 2:** Channel 1 Network Layout.

Layer	Patch size/Description	Output size	Parameters
Input	Integer Sequence	(batch, seq_len)	0
Embedding	vocab_size(256) $\rightarrow$ 128-dim	(batch, seq_len, 128)	vocab_size $\times$ 128
Multiscale Conv1D			
Path 1	1$\times$3	(batch, 128, seq_len)	49.4K
Path 2	1$\times$5	(batch, 128, seq_len)	82.3K
Path 3	1$\times$7	(batch, 128, seq_len)	115.2K
Dilated Conv Groups For $k=3,5,7$	$3\times [1\times 3, m=1,2,5]$	(batch, 128, seq_len)	443.5K
Adaptive MaxPool1d	Global pooling	(batch, 128, 1)	0
Residual Connection	-	(batch, 384)	49.5K
Feature Fusion	Concatenate	(batch, 1536)	0

### The coding algebraic-feature branch

For effective blind identification of unknown channel coding types, this branch extracts seven complementary features from received codeword sequences. These features comprehensively characterize sequence properties across time, statistical, structural, and frequency domains, providing discriminative information for classifier differentiation between coding schemes.

The run count quantifies basic sequence structure by counting identical-symbol runs. Its mathematical definition is

8 $$\begin{aligned} R(\textit{w}) = \Vert {\{ i \mid w_i \ne w_{i+1} \}} \Vert \ + 1 \end{aligned}$$

This feature sensitively identifies encodings introducing long “0” or “1” sequences–particularly Hamming and Polar codes.

Coding depth quantifies sequence algebraic complexity as the minimal order k where the k-th finite difference converges to zero, i.e.,

9 $$\begin{aligned} D(\textit{w}) = \min \{ k \mid \nabla ^{k} \textit{w} = \textit{0} \} \end{aligned}$$

This metric effectively distinguishes memory codes (higher k) from memoryless block codes (lower k).

Shannon entropy quantifies sequence randomness, where values approaching 1 indicate high randomization. Iteratively decoded codes (e.g., Turbo/LDPC codes) exhibit higher entropy post-decoding due to interleaving, while well-structured algebraic codes (e.g., BCH) yield lower values. We thus employ entropy metric to distinguish encoding randomization levels, i.e.,

10 $$\begin{aligned} H(\textit{w}) = -\dfrac{1}{L} \sum _{i=1}^{L} p_i \log _2 p_i \end{aligned}$$

Certain codes (e.g., convolutional codes and RS codes) introduce distinct periodicity, generating pronounced peaks in the autocorrelation function. We employ sequence autocorrelation to identify such periodic structures,

11 $$\begin{aligned} A_{cc}(k) = \dfrac{1}{L-k} \sum _{i=1}^{L-k} w_i w_{i+k} \end{aligned}$$

where $A_{cc}(k)$ measures correlation between a sequence and its k-lag shifted version.

To characterize fine-grained local statistics in long input sequences, we extract block features by segmenting sequences into fixed-length sub-blocks. For each block ${b_j}$ of size *l*, we compute block weight, entropy and variance to effectively reflect local correlations determined by encoding structures, i.e.,

12 $$\begin{aligned} \textrm{weight}^{\ell }= & \operatorname {Var}\left( \sum _{i \in \textit{b}_j} b_i \right) ^{\!L/l} \end{aligned}$$

13 $$\begin{aligned} \textrm{entropy}^{\ell }= & \frac{1}{L/l} \sum _{j=1}^{L/l} H(\textit{b}_j) \end{aligned}$$

14 $$\begin{aligned} \textrm{variance}^{\ell }= & \operatorname {Var}\left( \operatorname {Var}(b_j) \right) ^{(L/l)} \end{aligned}$$

To enhance differentiation among linear block codes with limited intrinsic discriminability, we introduce linear features detecting potential linear constraints, including linear correlation and parity-check mean, i.e.,

15 $$\begin{aligned} C_{\textrm{lin}}(\textbf{w})= & \frac{1}{20} \sum _{i=1}^{20} P(w_{a_j} = w_{b_j}) \end{aligned}$$

16 $$\begin{aligned} P(\textbf{w})= & \frac{1}{10} \sum _{j=1}^{10} \left( \sum _{i \in I_j} w_i \mod 2 \right) \end{aligned}$$

where $P(\cdot )$ is the indicator function, $a_j, b_j$ are random bit positions, and $I_j$ denotes random size-j subsets. The former estimates linearity by evaluating the consistency of randomly sampled bit pairs, while the latter directly simulates the parity-check process. Lower parity-check mean values (approaching 0) indicate higher likelihood of satisfying linear system parity equations.

Different encoding schemes produce distinct spectral signatures due to structural differences. We compute spectral features via discrete Fourier transform, including mean amplitude, spectral variance and maximum amplitude, i.e.,

17 $$\begin{aligned} M= & \dfrac{1}{5} \sum _{w \in \Omega } |\mathscr {F} (\textit{w}) |_{m} \end{aligned}$$

18 $$\begin{aligned} V= & \operatorname {Var}( |\mathscr {F}(\textit{w}) |) \end{aligned}$$

19 $$\begin{aligned} M_{\text {max}}= & \max ( |\mathscr {F}(\textit{w}) |) \end{aligned}$$

where $\mathscr {F}(\textit{w})$ denotes codeword spectrum. These metrics enable frequency-domain differentiation of encoding types.

The seven features comprehensively cover time-domain (run count, autocorrelation), statistical (entropy, block features), structural (coding depth, linear features), and frequency-domain (spectral features) characteristics, preventing critical discriminative information loss from single-domain analysis. Each feature exhibits orthogonal sensitivity to specific encoding types, collectively spanning a highly discriminative feature space for effective encoding separation. Moreover, the artificial features’ low BER sensitivity enhances overall network robustness.

**Fig. 4:**
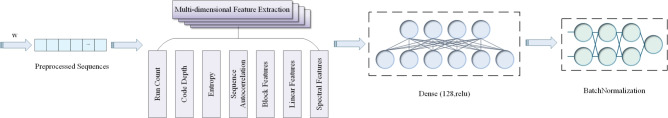
The structure of algebraic-feature branch.

As depicted in Fig. 4, the multi-dimensional feature extraction module takes the preprocessed sequence as input and produces a high-dimensional discriminative feature tensor via the feature space mapping. This tensor is formally expressed as

20 $$\begin{aligned} \textbf{F}_{\textrm{multi}} = T\left( X_{\textrm{pre}} \right) \in \mathbb {R}^{D} \end{aligned}$$

where $T(\cdot )$ denote the feature-transformation operator and *D* the resulting feature dimension. The obtained tensor $\textbf{F}_{\textrm{multi}}$ is forwarded to the downstream network, where a fully-connected layer performs dimension upscaling and nonlinear mapping. The GELU activation is adopted, and the weight matrix $\textbf{W}_{f} \in \mathbb {R}^{128 \times 7}$ is initialized via the Xavier scheme with bias vector $\textbf{b}_{f} \in \mathbb {R}^{128}$.

21 $$\begin{aligned} \textbf{h}_{fc} = \operatorname {GELU}\left( \textbf{W}_f \textbf{F}_{\text {multi}} + \textbf{b}_f \right) \in \mathbb {R}^{128} \end{aligned}$$

Subsequently, a batch-normalization layer followed by 50% dropout is applied to enhance model generalization. The specific network distribution of the coding algebraic-feature branch is shown in Table 3.

**Table 3:** Channel 2 Network Layout.

Layer	Patch size/Description	Output size	Parameters
Input	Handcrafted features	(batch, num_features)	0
FC	num_features $\rightarrow$ 256	(batch, 256)	num_features $\times$ 256 + 256
Activation	RELU	(batch, 256)	0
BatchNorm	-	(batch, 256)	512
Dropout	rate = 0.5	(batch, 256)	0

## Experimental results

### Experimental platform and dataset setup

All experiments were conducted with the PyTorch deep-learning framework and accelerated on an NVIDIA GeForce RTX 4060 GPU. To satisfy the data demands of the network, a synthetic dataset comprising seven channel-coding types–Hamming, BCH, LDPC, RS, Polar, convolutional, and Turbo–was generated in MATLAB 2022a. For each parameter setting listed in Table 4, 2,000 frames were produced, yielding 1,120,000 samples after adding noise at twenty BER values uniformly spaced in the interval $10^{-5}\sim 10^{-1}$. Each code type contains $4 parameter sets\times 20 BER points\times 2000 samples=1.6\times 10^{5} samples$, giving a grand total of $7 types\times 1.6\times 10^{5} = 1.12\times 10^{6} samples$. The dataset is split 8:2 by independently drawing different Monte-Carlo runs: the training set comprises samples generated from the first 80% of random-information runs, while the test set exclusively uses the remaining 20%, guaranteeing mutually independent generation and eliminating any structural leakage.

Since the paper targets blind identification on the bit stream after demodulation, all physical channel impairments are condensed into bit errors. We therefore use BER as a unified and sufficient impairment metric and inject errors via reproducible Monte-Carlo bit flipping: For every stored codeword $C \in \{0,1\}^{n}$ (n=5000 bits), we draw an independent error pattern $E \in \{0,1\}^{n}$ whose entries are i.i.d. Bernoulli($p=BER_target$); The outer Monte-Carlo loop resets rng(2022+i) before any noise generation, guaranteeing reproducibility.

500 independent trials were executed, each with a re-seeded random number generator for dataset splitting, PyTorch weight initialization, and BER noise injection. Mean and standard-deviation results reported in the paper are computed over these 500 runs.

Model training employed the Adam optimizer with an initial learning rate of $10^{-3}$ and decay factors $beta\_1=0.9,beta\_2=0.999$. The cross-entropy loss $\mathscr {L} = -\sum _{k=1}^{7} y_{k} \log \hat{y}_{k}$ served as the objective function; the batch size was fixed at $B=256$ and training was terminated after $T=150$ epochs.

To mitigate overfitting, an early-stopping criterion is implemented: training is automatically terminated if the validation loss has not improved for 15 consecutive epochs. In addition, a dynamic learning-rate scheduler is employed that halves the learning rate whenever the validation loss plateaus for 5 epochs, subject to a lower bound of $10^{-6}$.

**Table 4:** Code parameters description.

Code type	Code parameters
Convolutional Codes	(4,[15,17]) (5,[23,35]) (6,[53,75]) (7,[133,174])
Hamming Codes	[7,4] [15,11] [31,26] [63,57]
LDPC Codes	[648,1/2] [648,2/3] [1296,1/2] [1296,2/3]
Turbo Codes	(4,[15,17]) (5,[23,35]) (6,[53,75]) (7,[133,174])
BCH Codes	[63,47] [127,95] [204,188] [255,223]
RS Codes	[15,7] [15,11] [255,139] [255,251]
Polar Codes	[64,32] [128,40] [256,80] [512,256]

### Network performance analysis under different conditions

Beyond hyperparameter selection, training parameter configuration and dataset design, the performance of DBFCNN can also be significantly influenced by factors such as network architecture, embedding dimension, and sequence semantic segmentation length. Comprehensive consideration of these factors is essential during model design. This section investigates the impact of these architectural and representational parameters on model performance.

#### Comparison of recognition performance between different models

**Fig. 5:**
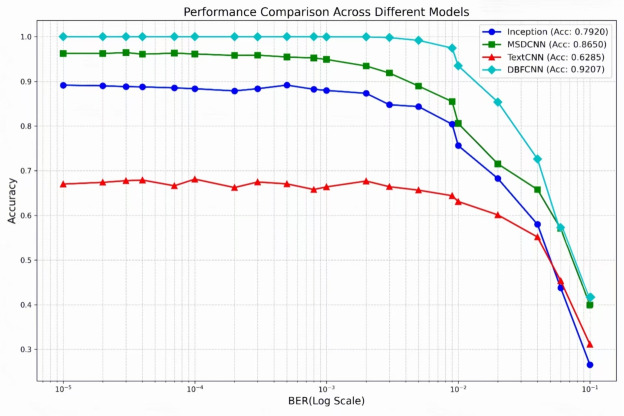
Performance comparison of DBFCNN with others.

We evaluated the performance of DBFCNN against several established models under varying BERs. The comparative models included: MSDCNN^24^, employing dual multi-scale modules and dilated convolutions; TextCNN^26^, which processes encoded sequences by analogy to textual data; and a fusion Inception network^35^ comprising initial dual convolutional layers followed by five multi-scale Inception modules. Experimental results, presented in Fig. 5, demonstrate that DBFCNN achieves significantly superior performance across the examined BER spectrum. Notably, at low error rates ($10^{-5}\sim 10^{-3}$), DBFCNN maintains an accuracy exceeding 98%.

Among the reference models, MSDCNN achieved the highest baseline performance, yet its recognition accuracy remained substantially lower than that of DBFCNN. At BERs of $10^{-5}$, $10^{-4}$, $10^{-3}$, $10^{-2}$ and $10^{-1}$, DBFCNN improved recognition accuracy by 3.75%, 3.86%, 5.05%, 12.91%, and 0.78%, respectively, over MSDCNN. This superiority is primarily attributable to DBFCNN’s dual-branch feature fusion mechanism, which enhances feature representation capabilities and model robustness in complex channel environments. Experimental results further demonstrate that in high-BER regions ($BER\ge 10^{-2}$), DBFCNN maintains an average performance advantage of 82.30%.

#### The impact of semantic segmentation length on performance

**Fig. 6:**
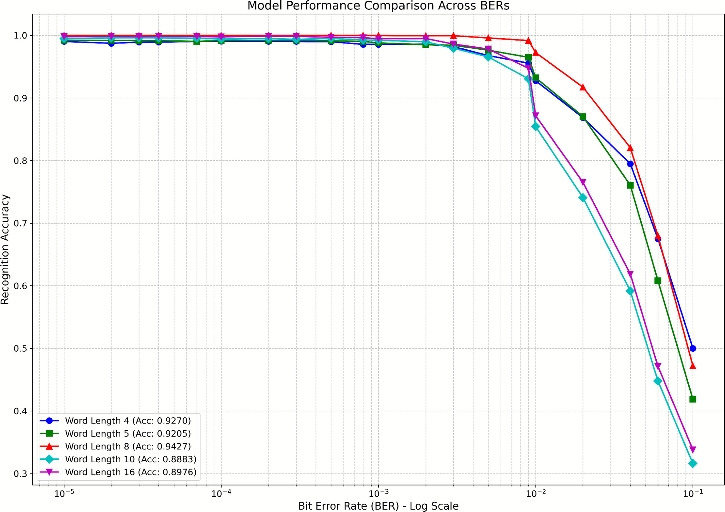
Recognition accuracy of DBFCNN for different word length.

Figure 6 presents a comparison of recognition results for several common semantic semantic divisions with lengths of $g=\{4,5,8,10,16\}$ bits. Experimental results indicate that a word length of 8 bits achieves the highest average recognition accuracy of  94.27% (±0.3%) across the entire BER range $10^{-5}$ to  $10^{-1}$. This optimality arises because the 8-bit length aligns with 1-byte memory cells, allowing single-cycle SIMD loads and reducing memory transactions by 50% compared to 16-bit words. Critically, an 8-bit window effectively encompasses the core structural parameters of major channel coding schemes: the 5–7 bit constraint length of convolutional codes, the byte-oriented interleaver design of Turbo codes, the native 8-bit symbol system of RS/BCH codes, and the byte-aligned parity-check matrix structure typical of LDPC codes.

However, as shown in Fig. 6, under high BER conditions ($BER> 6\times 10^{-2}$), 8-bit grouping exhibits 1.26% lower recognition accuracy (61.10% vs. 62.36%) compared to 4-bit grouping. This occurs because shorter word lengths partition sequences into more fragments with reduced information per word, thereby diminishing single-bit error impacts. Conversely, 8-bit words carry higher information density, making single-bit errors more detrimental to feature vectors. Consequently, 4-bit grouping achieves superior accuracy at high BER. Nevertheless, since operational BER operational BER is typically $< 6\times 10^{-2}$ in practical communications, 8-bit grouping remains optimal overall.

#### Impact of network architecture on performance

**Fig. 7:**
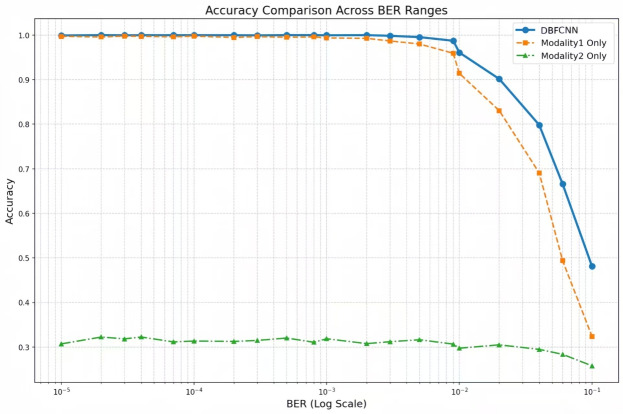
Recognition accuracy of DBFCNN for different model frame.

**Fig. 8:**
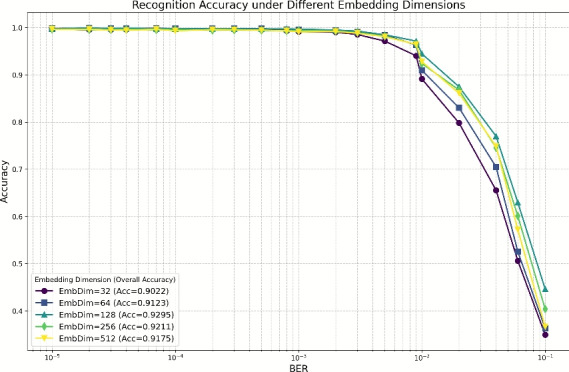
Recognition accuracy of DBFCNN for different embedding dim.

**Fig. 9:**
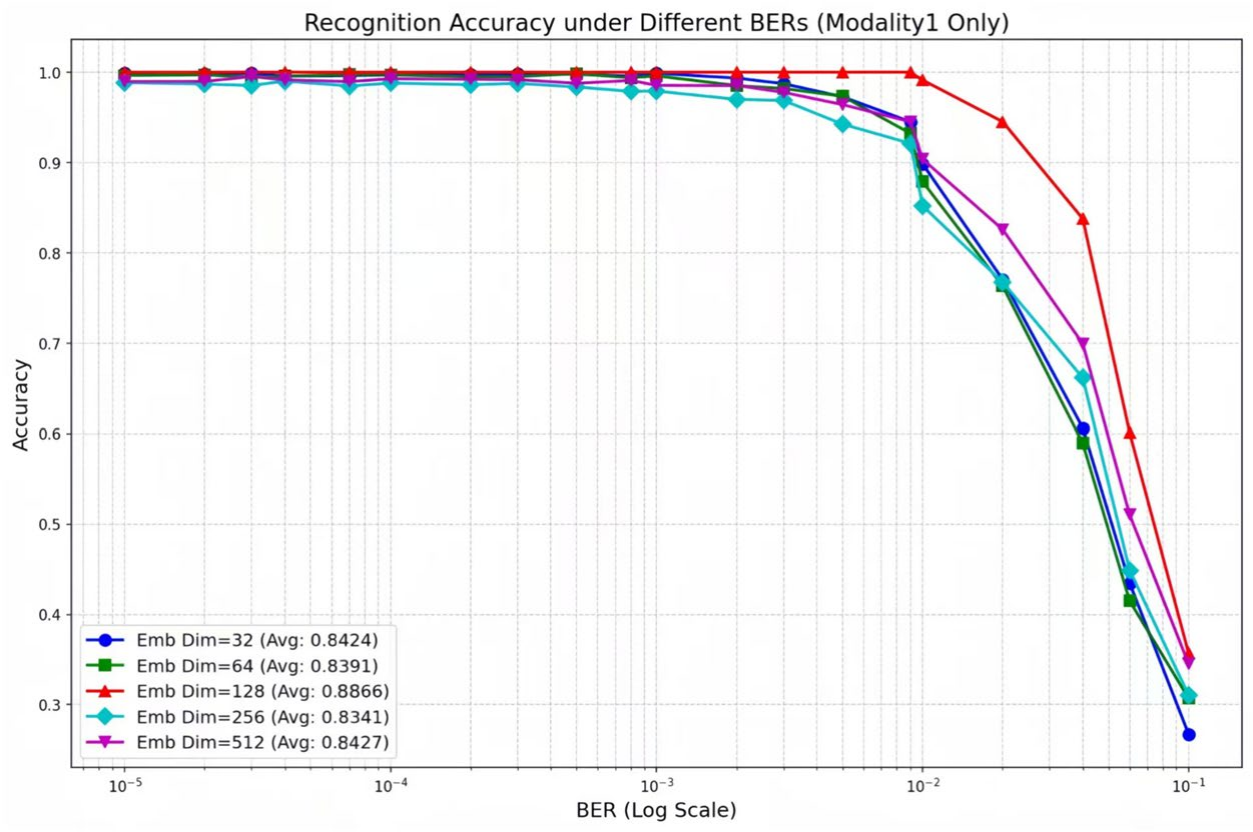
Recognition accuracy of modality1 for different embedding dim.

Figures 7, 8 and 9 present recognition performance comparisons across diverse network architectures and embedding dimensions. Experimental results demonstrate that the DBFCNN achieves peak performance with an embedding dimension of 128, attaining a test accuracy of 92.95%. This represents a 4.29 percentage point improvement over the optimal single-branch architecture. A dimension of 128 aligns with typical GPU memory bus and cache line sizes, minimizing memory overhead. Besides, Fig. 8 shows 128 sits at the inflection point–32/64 under-fit (90%), while 256/512 over-fit (92.11%/91.75%). This matches the bias-variance trade-off. Moreover, 128 provides sufficient capacity for both raw-sequence and handcrafted-feature branches before fusion, avoiding information bottlenecks (Fig. 9, single-modality: 88.66% at 128). Thus, 128 is not empirical-only but arises from computational alignment, bias-variance balance, and systematic ablation.

Crucially, as embedding dimension increases from 32 to 512, DBFCNN exhibits a standard deviation of 2.73 % (compared with 5.25% for the optimal single-branch network) compared to the optimal single-branch architecture (5.25%). This 2.52-percentage-point reduction in standard deviation confirms that the dual-branch design mitigates embedding-dimension-induced accuracy swings by 48%. The enhanced stability originates from the dual-branch feature fusion mechanism, which optimizes representation space enhancement at the 128-dimension configuration.

As evidenced in figure 7, DBFCNN maintains maintains an average accuracy $\ge 91.17\%$ over the entire $10^{-5}$ to  $10^{-1}$ BER range, outperforming the single-branch counterpart by at least 0.26 percentage points at every BER level. This is because the dual-branch design synergistically combines convolutional networks’ nonlinear discriminative power with handcrafted features’ BER robustness, overcoming single-modality limitations through complementary fusion, enhancing recognition performance and stability in dynamic channel environments.

#### Ablation study on the efficacy of the semantic branch

**Fig. 10:**
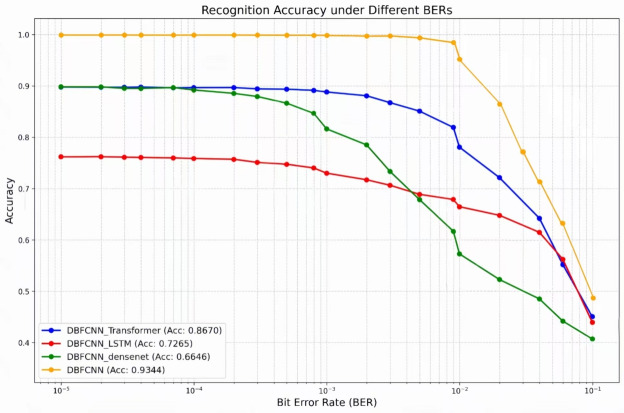
Recognition performance of DBFCNN for replaced branch 1.

To verify that the recognition performance of DBFCNN primarily stems from the structural design of its semantic channel for encoded sequences (branch 1), we replaced branch 1 with several high-performance network architectures–including DenseNet121 (with residual connections), LSTM (with gating mechanisms), and Transformer (with self-attention for global dependency modeling)–while retaining the algebraic feature channel (branch 2). This setup allowed us to evaluate the classification capabilities of different networks on encoding types. Experimental results are shown in Figure 10.

Experimental results demonstrate that the DBFCNN framework achieves the highest classification accuracy across all tested bit error rate conditions, outperforming architectures where branch 1 is replaced by DenseNet121, LSTM, or Transformer. These findings confirm the critical role of the structural design of the semantic branch (branch 1) in DBFCNN’s recognition performance. The multi-scale dilated convolution module effectively captures discriminative features from encoded sequences, while alternative architectures–despite their strong general representation capabilities–fail to encode the structural patterns specific to channel coding with equal efficacy. Thus, the specialized design of the semantic branch is the principal factor underlying DBFCNN’s superior performance, rendering it well-suited for encoding recognition in complex channel environments.

#### Identification of different encoding types

**Fig. 11:**
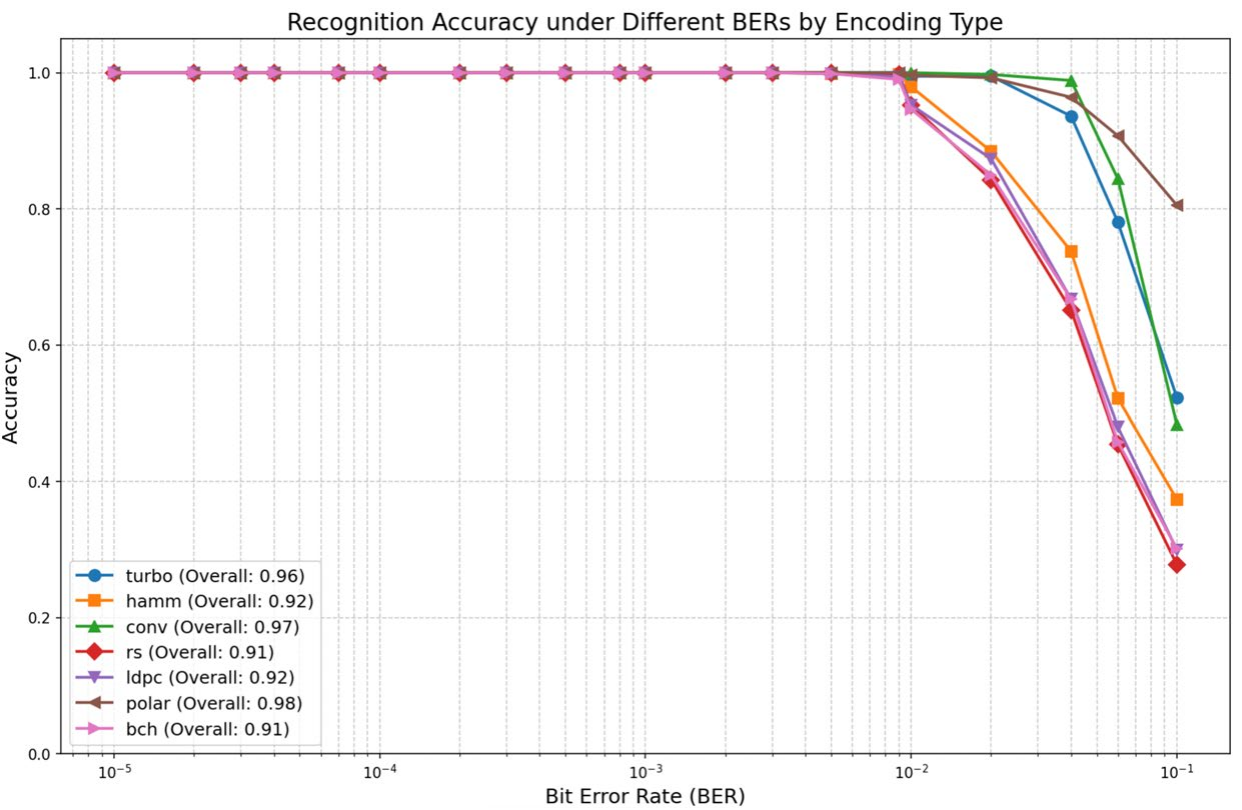
Recognition accuracy of DBFCNN for different type.

Figure 11 illustrates the recognition performance variation across channel coding types as a function of BER. Hamming codes–the simplest linear block codes–exhibit stronger recognizability at lower BERs. However, recognition performance degrades for more complex linear block codes under identical conditions. In contrast, convolutional codes, Turbo codes, and Polar codes demonstrate significantly superior recognition capabilities at elevated BERs. Notably, Polar codes maintain $> 80\%$ recognition accuracy even at $BER=10^{-1}$. Within medium-to-low BER regimes ($10^{-5}\sim 10^{-3}$), all coding schemes achieve $> 95\%$ recognition accuracy, asymptotically approaching 99% as BER decreases further.

**Fig. 12:**
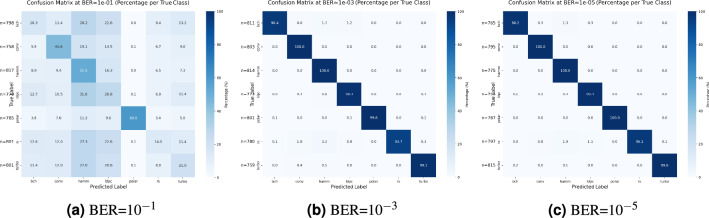
Confusion matrices of DBFCNN trained on the simulation dataset for different BER values.

To delineate performance variations across channel coding schemes, Fig. 12a to Fig. 12c present confusion matrices under high ($BER=10^{-1}$), medium ($BER=10^{-3}$), and low ($BER=10^{-5}$) BER conditions. Under severe noise (Fig. 12a), Polar codes maintain 60% recognition accuracy attributed to identifiable frozen-bit patterns from channel polarization, while Hamming codes achieve 51.5% accuracy through their fixed grouping structure; conversely, other types of codes exhibit significant cross-confusion. In medium-error environments (Fig. 12b), convolutional, Hamming, and polar codes attain perfect classification, whereas RS codes misclassify Hamming codes at 3.3% due to similar algebraic constructions, and LDPC codes show minimal confusion (0.8%) from sparse parity-check feature overlaps. Under pristine channel conditions (Fig. 12c), all schemes exceed 99% average accuracy with residual errors confined to BCH to Hamming misclassification (1.3%) from cyclic polynomial similarities and RS to convolutional/Hamming errors (2.7%) attributable to Galois field operation interference.

**Fig. 13:**
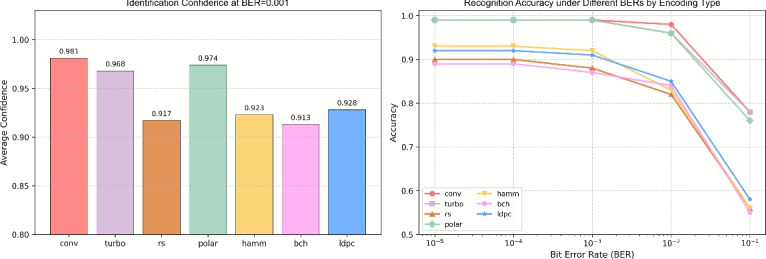
Recognition accuracy of same information using different channel codings and BERs.

To intuitively demonstrate performance, we adopts a “same source, multiple codes, multiple BERs” comparison. Generate 200 random bit sequences as the original source, encode each with seven target codes (Hamming, BCH, LDPC, RS, Polar, convolutional, Turbo), and fix the length at 5000 bits. Then inject the BER levels mentioned above into each encoded bit stream to obtain error-corrupted received sequences. Feed these erroneous bit streams into the DBFCNN identifier, which outputs per-code confidence (Fig. 13 left) and recognition-accuracy curves for the same source under different codes and BERs (Fig. 13 right). Experimental results shows that at $BER=10^{-3}$, confidence $\ge 88\%$ for all seven codes; at $BER=10^{-1}$, LDPC, Polar, convolutional and Turbo remain $\ge 80\%$, confirming effectiveness under strong noise.

#### Experimental validation of multi-scale feature extraction effectiveness

This section aims to validate the effectiveness of the proposed multi-scale convolutional neural network architecture. Experiments were conducted using the same training and testing datasets to compare the complete model (containing three branches with kernel sizes 3, 5, and 7) against individual single-branch variants. All experiments were performed under identical hardware environments and hyperparameter settings to ensure result comparability.

**Fig. 14:**
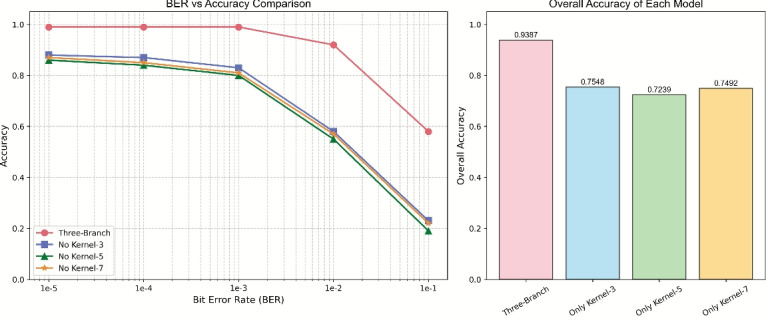
Accuracy performance of multi-scale and single-branch models.

As shown in Fig. 14, the complete multi-branch model achieved an average recognition accuracy of 93.95% on the test set, significantly outperforming any single-branch variant. Specifically, single-branch models using only kernel sizes 3, 5, and 7 achieved accuracies of 89.32%, 88.40%, 89.15% respectively, indicating that multi-branch collaboration improves recognition performance by approximately 4.6–5.6 percentage points.

Convolutional kernels of different scales demonstrate clear complementary characteristics in feature extraction. Small-scale kernels (size=3) excel at capturing local details and short-range dependencies but have limited perception of long-range correlations; large-scale kernels (size=7) are adept at capturing global structural features but are slightly inferior in fine-grained feature extraction; medium-scale kernels (size=5) are between the two.

**Fig. 15:**
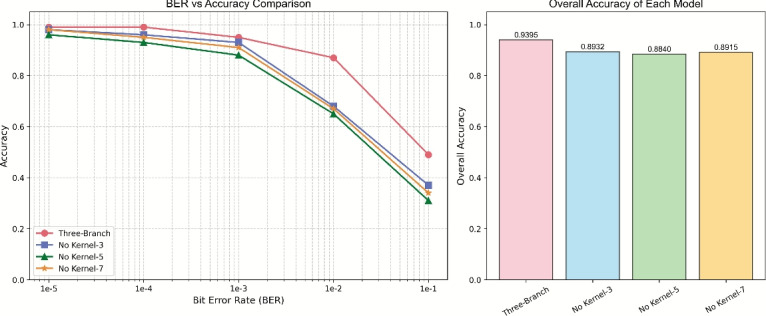
Accuracy performance of multi-scale and dual-branch models.

As shown in Fig. 15, systematic removal of individual branches revealed significant performance degradation. Removing the size=3 branch reduced local feature extraction capability, decreasing accuracy by 4.63 percentage points; removing the size=5 branch impaired medium-range context modeling, decreasing accuracy by 5.55 percentage points; removing the size=7 branch weakened global structure perception, decreasing accuracy by 4.80 percentage points. These results fully demonstrate the necessity of multi-scale feature complementarity.

Under different BER conditions, the complete model consistently demonstrated the best robustness. Particularly in high BER ($BER\geq10^{-2}$) environments, the complete model maintained recognition accuracy above 0.8, while all single-branch models experienced significant performance degradation, further validating the anti-interference capability of the multi-scale architecture.

### Comprehensive performance evaluation and analysis

**Fig. 16:**
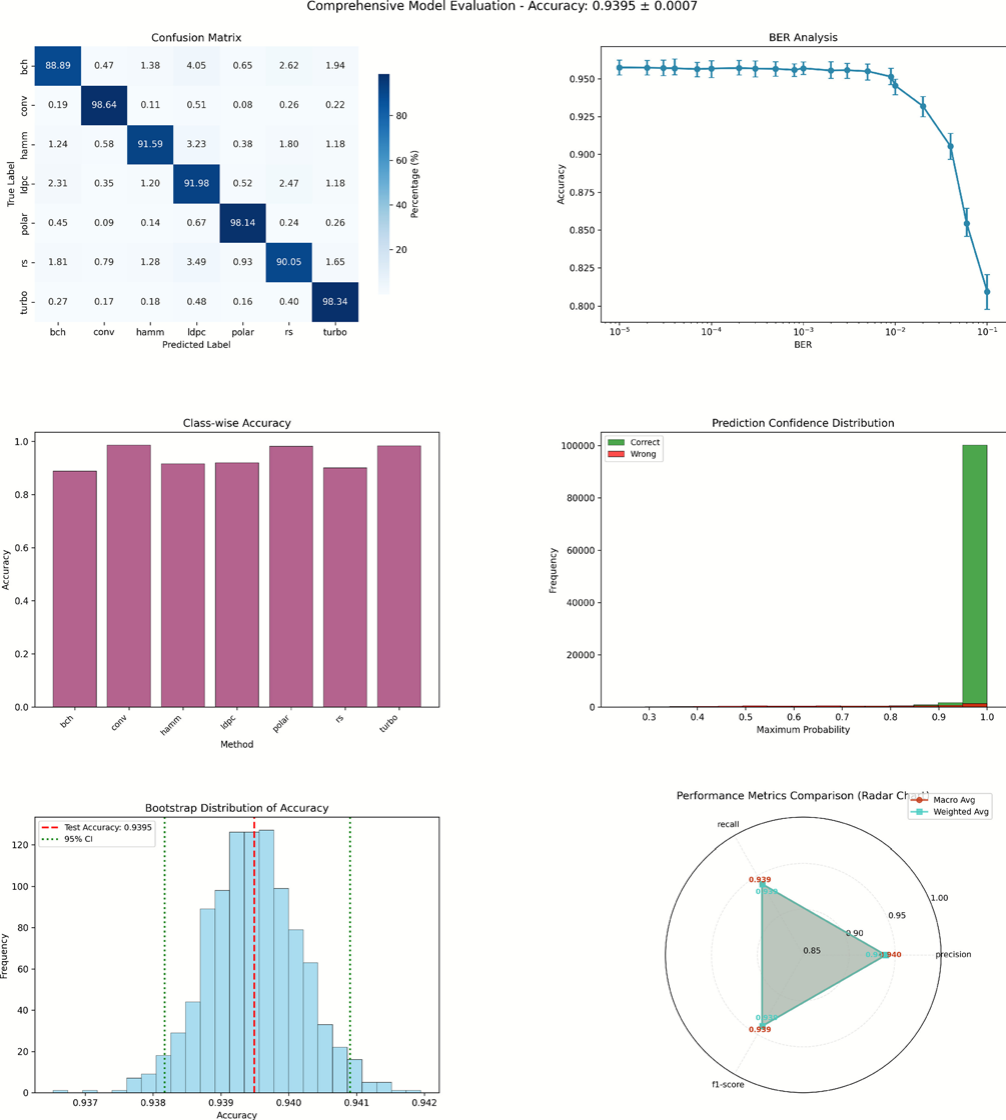
Comprehensive performance of DBFCNN.

As shown in Fig. 16, the model achieves an accuracy of 93.95% on the test set, with a standard deviation of only ±0.0007, indicating highly stable prediction performance. The 95% confidence interval of [0.937, 0.942] further validates the statistical reliability of the results.

The model exhibits strong class-wise performance, with the confusion matrix showing high accuracy across all encoding types, notably convolutional and Turbo codes achieving over 96% accuracy, demonstrating effective differentiation of encoding structures. Class accuracy is balanced, with F1-scores consistently above 0.9, supported by a uniform dataset distribution for reliable evaluation. In BER robustness tests, accuracy declines with increasing error rates but remains above 85% at high BER ($10^{-2}$) and stable at 90–93% in typical scenarios ($10^{-3}$ to $10^{-4}$). Statistical validation confirms significance through a concentrated Bootstrap distribution with low standard deviation, and consistency between macro and weighted averages underscores performance stability.

## Conclusion

To strengthen autonomous sensing in non-cooperative scenarios, we propose DBFCNN, a neural network that embeds coding-domain knowledge into deep learning so that secondary users can identify the primary user’s channel-code type without any prior information. A TextCNN-based approach employing dilated convolution modules instead of standard 1D convolutions is first introduced. By implementing varying dilation rates, the network achieves multi-scale feature perception. Furthermore, both local patterns and global statistical features from raw bit sequences are utilized to provide complementary information for identifying different channel coding types. DBFCNN’s classification accuracy is evaluated across varying BER conditions. Experimental results demonstrate superior performance to TextCNN, Inception, and MSDCNN architectures at both low and high BER regimes. Overall accuracy improved by 5–10% versus state-of-the-art baselines. At $BER\le 10^{-3}$, test accuracy exceeds 98%.

However, performance degradation remains challenging at extremely high BER condition. Future work will investigate alternative architectures, streamlined feature extraction, and discriminative feature engineering for linear block codes to enable fine-grained recognition in high-BER regimes. Furthermore, we will consider an end-to-end model that jointly estimates frame boundaries and recognizes code types, to cope with possible sync errors and codeword offsets in practical cognitive-radio scenarios.

## Data Availability

The datasets used during the current study available from the corresponding author on reasonable request.
